# Analysis of Nutritional Characteristics and Willingness to Pay of Consumers for Dry-Cured Sausages (*Salchichón*) Made with Textured Seed Oils

**DOI:** 10.3390/foods12163118

**Published:** 2023-08-19

**Authors:** Laura Tarjuelo, Adrián Rabadán, Manuel Álvarez-Ortí, Arturo Pardo-Giménez, José E. Pardo

**Affiliations:** 1 Escuela Técnica Superior de Ingeniería Agronómica y de Montes y Biotecnología, Universidad de Castilla-La Mancha, Campus Universitario, s/n, 02071 Albacete, Spain; laura.tarjuelo@alu.uclm.es (L.T.); manuel.alvarez@uclm.es (M.Á.-O.); jose.pgonzalez@uclm.es (J.E.P.); 2 Centro de Investigación, Experimentación y Servicios del Champiñón (CIES), C/Peñicas, s/n, 16220 Quintanar del Rey, Spain; apardo.cies@dipucuenca.es

**Keywords:** unsaturated fatty acids, functional foods, chia oil, poppy oil, melon oil, pumpkin oil

## Abstract

The consumption of processed meat products beyond recommended limits has been associated with serious health conditions, including heart disease, diabetes, and cancer. In an effort to create healthier options, the meat industry is exploring alternatives to animal fat in processed meats. This study focuses on replacing animal fat in dry-cured sausages (*Salchichón*) with textured chia, poppy, melon, and pumpkin oils. The research aims to evaluate the physical and nutritional changes resulting from this substitution and assess consumer acceptance through sensory analysis. The use of seed oils led to slight color changes and comparable texture, except for cohesiveness. The incorporation of textured seed oils resulted in reduced fat content, increased proportions of ashes and protein, and decreased energy value. The fatty acid composition showed lower proportions of saturated fatty acids and increased polyunsaturated fatty acids. Sensory analysis revealed that the control sample with pork fat received the highest ratings for appearance, texture, and taste, while samples with higher seed oil percentages scored lower due to color, cohesiveness, and specific flavors from the seed oils. Despite these variations, consumers demonstrated a high level of acceptability for all samples. Choice analysis results indicated that higher prices had a negative impact on consumer willingness to purchase, while the use of the 100% Iberian pig breed and animal welfare labels positively influenced purchasing attitudes. Regarding the presence of a pumpkin seed oil label in the product, a negative willingness to pay was reported. However, significant individual variation was reported for this attribute, indicating the existence of consumer segments with more positive attitudes toward these innovative dry-cured sausages.

## 1. Introduction

The current diet in many developed countries includes processed meat products such as cured meats, which are popular and are part of people’s regular diet. These products are a source of proteins, B vitamins and minerals such as iron or zinc and can have beneficial effects on human health [1,2,3]. However, the consumption of processed meat products beyond recommended limits has been implicated in the development of several serious health conditions, including coronary heart disease, type 2 diabetes, and colon cancer [4]. The precise mechanisms through which these detrimental effects occur, particularly the relationship between processed meat and cancer, are not yet fully understood. However, the meat industry faces the challenge of replacing ingredients considered harmful with health-promoting alternatives in processed meats in order to make healthier products that meet the demands of consumers [5,6].

“*Salchichón*” is a traditional dry-cured sausage that is prepared by combining minced pork meat and fat, typically seasoned with spices, and optionally supplemented with a starter culture. Similar products are elaborated in different countries, like salami in Italy or saucisson in France [7]. During the curing process, the product undergoes biochemical, microbiological, and physical transformations that give rise to its distinctive flavor and texture while simultaneously extending its shelf-life [8,9]. It contains significant amounts of pork fat, which plays a crucial role in flavor development by facilitating flavor distribution and allowing spices to permeate the meat mixture. Furthermore, the fat contributes to the texture of the sausage, since it helps bind the meat particles together, enhancing cohesiveness. However, animal fat is often perceived as detrimental by consumers due to its elevated saturated fatty acid content. Consequently, the industry is actively striving to reduce the fat content in these products while also aiming to achieve a more balanced fatty acid profile [10,11].

For a few years now, the agri-food industry has been focusing on searching for new products that demonstrate a beneficial effect on the body’s functions, improving the health and well-being of individuals and reducing the risk of these diseases. In this sense, vegetable oils are a good option to include in meat products to replace animal fat, since they are rich in polyunsaturated fatty acids, vitamin E and bioactive substances with antioxidant capacity [12]. The oils obtained from chia, poppy, pumpkin or melon seeds are distinguished by their substantial content of polyunsaturated fatty acids, notably essential fatty acids such as linoleic (ω-6) and linolenic (ω-3) acid that must be included in the human diet since they cannot be endogenously synthesized, playing a crucial role in the synthesis of long-chain polyunsaturated fatty acids involved in many metabolic processes related to the reduction in oxidation and inhibition of inflammation [13].

Chia oil has emerged as a prominent functional food in recent times due to its remarkable health benefits attributed to its unique chemical composition. This oil is particularly rich in α-linolenic acid (ω-3), which can reach about 60% of their fatty acids [14], but it also contains tocopherols, phenolics and other bioactive compounds with antioxidant capacity [15,16]. On the other hand, poppy, melon, and pumpkin seed oils contain a major proportion of linoleic acid (ω-6). In the case of poppy seed oil, the content of linoleic acid exceeds 70% [17], while in melon or pumpkin seed oils, this content usually represents between 40 and 60% [18,19]. The high content of unsaturated fatty acids in these oils gives them a liquid consistency at room temperature. Therefore, when using them as substitutes for animal fats, it is necessary to modify their texture to achieve similar characteristics to animal fats. There are various strategies to achieve a solid or semi-solid texture in vegetable oils, such as pre-emulsification, microencapsulation, the formation of oleogels, or the development of gelled emulsions [20]. By employing emulsifiers or gelling agents, it is possible to obtain a product with vegetable oils rich in unsaturated fatty acids with a stable semi-solid texture that can replicate the characteristics of animal fat [21].

Although food innovation is crucial to ensure the production of healthier and more sustainable foods, the consumer perspective must be always considered [22]. Although some consumers are open to try novel foods, others tend to reject new foods or foods that include new or unconventional ingredients [23]. The so-called food neophobia varies depending on the consumers’ personal characteristics, knowledge, attitudes, or motivations [24]. Moreover, consumers decide the purchase of a product by analyzing the available information in the moment of the purchase [25] with food labeling being key in determining consumers’ attitudes [26]. In this regard, discrete choice models are useful as they represent the choice situations that consumers face in real life [27].

The aim of this study is to partially or completely replace the animal fat usually employed in dry-cured sausages (*Salchichón*) production with textured chia, poppy, melon, and pumpkin oils. The variations in product characteristics will be assessed from both a physical and nutritional perspective. Finally, a sensory analysis will be conducted to determine consumer acceptance.

## 2. Materials and Methods

### 2.1. Ingredients and Elaboration of Dry-Cured Sausages

The ingredients included in the formulation of the dry-cured sausage were lean pork meat and pork fat as well as oil from chia, poppy, melon, and pumpkin seeds, which were textured with inulin and guar gum along with other condiments. The pork meat and fat, the chia and poppy seeds and the condiments (salt and spices) were purchased from local markets. The texturizers (inulin and guar gum) were obtained from Sosa Ingredients S.L. (Barcelona, Spain). Finally, the melon and pumpkin seeds were collected from the IV range industry Vicente Peris S.A. (Albuixet, Valencia, Spain), where these seeds are considered as waste from their production.

The oils from the four different seeds were extracted in a hydraulic press (MECAMAQ model DEVF 80) to obtain high-quality virgin oils [28]. Prior to extraction, the seeds were ground in a knife mill (RETSCH model GM 20). The resulting paste was then subjected to a pressure of 200 bar for 10 min. Finally, the obtained oil was centrifuged to remove any solid residue. The oils were stored in dark glass bottles at 4 °C until the dry-cured sausages were prepared. To achieve the correct texture of the oils to incorporate them into the sausage’s formulation, they were textured with guar gum and inulin mixing 210 g of seed oil, 14 g of guar gum, 21 g of inulin and 455 g of water. The mixture was stirred at high speed to form an emulsion, and then, it was stored under refrigeration to obtain a semi-solid texture.

To prepare the dry-cured sausages, the proportion of 76% of lean pork meat and 24% of fat was used. The control group consisted of sausages made with pork fat, while in the rest of the samples, the fat was partially substituted (50% and 75% of textured seed oil) or totally (100%). The mixing and the stuffing of the mixed ingredients into the casing were performed at the facilities of the meat industry El Conchel (El Ballestero, Albacete, Spain). The sausages were cured at 12 °C with a humidity level of 60% for 21 days. After this period, to prevent oxidation, the dry-cured sausages were stored under vacuum at 4 °C for a period of less than one week until analysis. All the samples were prepared in the same manner to ensure uniform shape. Nevertheless, at the conclusion of the curing process, a slight contraction can be observed in the samples where animal fat was completely replaced with textured vegetable oils due to an increased water loss during the process. This characteristic was evaluated in the sensory analysis under the parameter of external appearance.

### 2.2. Physical Measurements

The color of the samples was measured using a Minolta CR-300 colorimeter (Minolta Camera Co., Ltd., Osaka, Japan) using the illuminant D65. Color parameters (L*, lightness; a*, red–green component; b*, yellow–blue component; and C*: chroma) were measured in five slices from each sample.

To evaluate the texture of the different samples, a texture profile analysis test (TPA) was performed, using a texture analyzer TA-XT Plus (Stable Micro Systems, Godalming, UK) equipped with a 50 mm diameter probe at a speed of 3.3 mm/s. The analysis was performed at room temperature, and the slices were compressed to 60% of their original height. Hardness, springiness, cohesiveness, and chewiness were annotated in 5 different slices of each batch.

### 2.3. Proximate Analysis

The proximate analysis included the measurement of ash, protein, total fat, total carbohydrates and energy value according to official methods. All measurements were conducted on a dry basis. The ash content was determined by calcinating the samples at 550 °C until reaching a constant weight [29]. Fat content was measured by the filter bag technique after extraction with petroleum ether in an Ankom XT10 extraction system [30]. To evaluate the protein content, the nitrogen content was first measured using the Kjeldahl method, and then it was multiplied by a conversion factor of 6.25 [31]. Finally, the total carbohydrate content was calculated by subtracting the sum of the protein, fat, and ash contents from the total weight of the sample [32].

The energy value was estimated from the relative contents of the protein, fat and carbohydrates, applying Atwater factors of 4.0 kcal/g for protein, 9.0 kcal/g for fat and 4.0 kcal/g for carbohydrates.

### 2.4. Fatty Acids

To analyze the fatty acid content, the lipid fraction of the samples was derivatized to methyl esters and analyzed in a GC-2010 Plus gas chromatograph (Shimadzu, Tokyo, Japan) equipped with an automatic sampler, a split/splitless auto-injector (AOC-20i Shimadzu) and a Flame Ionization Detector (Shimadzu, Tokyo, Japan) [33]. A CP-Sil 88 silica capillary column (50 m × 0.25 mm inner diameter, 0.2 μm film thickness, Varian, Middelburg, Netherlands) was used with helium as the carrier gas at a flow rate of 3 mL/min.

The temperature program consisted of an initial hold at 120 °C for 5 min, which was followed by a gradient increase of 2 °C/min until reaching 160 °C, which was held for 2 min, and then a further gradient increase of 2 °C/min until reaching 220 °C, which was held for 10 min. Fatty acids methyl esters (FAMEs) were identified by comparing their retention times with a FAME 37 standard mix (Supelco, Bellefonte, PA, USA).

### 2.5. Sensory Analysis

To assess consumer acceptance and measure the subjective reaction to the product, a sensory affective test was chosen. Thus, a Measurement of Satisfaction Degree Test was designed with key-labeled samples, which were evaluated by 104 consumer-judges into a 9-point hedonic scale (−4: strongly dislike, 0: neither like nor dislike, +4: strongly like) [34]. The parameters evaluated were external appearance, texture and taste.

Sensory analysis was conducted in 2 tasting sessions to prevent judges from experiencing taste fatigue. In the first one, dry-cured sausages elaborated with textured oils from poppy and chia were tested, while in the second session, samples made with pumpkin and melon oils were tasted. Both sessions included a control sample elaborated with 100% pork fat.

### 2.6. Consumer Acceptance of the Dry-Cured Sausages

#### 2.6.1. Choice Set Design

A choice experiment was used to evaluate consumers’ attitudes toward the use of a specific seed oil (pumpkin oil) in the elaboration of the sausages. In this regard, a label was designed to inform consumers about the use of this oil in sausage reformulation (“with pumpkin seed oil”). Additionally, another three attributes were included: namely, price, pig breed and animal welfare certificate (Table 1). Traditionally, price is one of the attributes that has the greatest impact on consumers’ purchasing decisions [35]. The animal breed or the animal welfare certificate have also been previously identified as significant attributes in determining consumers’ attitudes toward pig products [36]. For the “price” attribute, three levels were chosen, reflecting the highest and lowest price at which a sausage can be found in Spain as well as the average of the two.

The four selected attributes, along with their various levels, were utilized to create 8 choice sets using the JMP program (JMP Statistical Discovery LLC, Cary, NC, USA). Each choice set presented to consumers consisted of two product alternatives and an option to opt-out. To mitigate the potential impact of presentation order on consumer choices, the choice sets were randomly presented to consumers [37].

#### 2.6.2. Replies

Surveys were conducted in person with individuals who had participated in the sensory evaluation of the product (104 consumers). This included students, faculty members, administrative and support staff, as well as other visitors to the Albacete university campus in Spain. The survey collected basic information such as gender, income, and level of education. Among the respondents, female consumers accounted for 62% of the sample, while males represented 38%. Approximately 46% of the participants in the sample were below the age of 29 (23% of them were over 50 years old), and up to 46% of them had completed university studies. Given the nature of the study and its location, it is expected that the sample is biased toward young and highly educated individuals, specifically those with university degrees. Similar characteristics can be observed in samples obtained through online sampling methods [38]. It is important to acknowledge this limitation when interpreting the results.

#### 2.6.3. Econometric Analysis

Consumer attitudes toward the purchase of burgers made with seed oil were examined using a discrete choice model (DCM). These models are built upon the concept of “Utility”, which refers to the net benefit that consumers derive from selecting a specific product in a choice situation involving various products that differ in certain attributes. Among the available DCMs, we employed the Mixed Logit (ML) model due to its flexibility and ability to adapt to real-world purchasing scenarios [39]. The model incorporated four selected attributes. PRICE was treated as a continuous variable with three levels (5.50; 8.00; 10.50 €), while the other variables (animal welfare, ANI.WELFARE; presence of seed oils, SEEDS; pork breed, BREED) were included as binary levels (presence and absence). The ML model was estimated using the Stata module mixlogit [40] run in STATA 17 software (Stata-Corp LP, College Station, TX, USA).

The ML model of utility for a sausage *j* for consumer *i* at choice occasion *t* is as follows:

$$U_{ijt}=\beta_{1i}PRICE_{ijt}+\beta_{2i}ANI.WELFARE_{ijt}+\beta_{3i}SEEDS_{ijt}+\beta_{4i}BREED_{ijt}+\varepsilon_{ijt}$$

### 2.7. Statistical Analysis

The data from the physico-chemical and sensory analysis were analyzed using a statistical approach to determine significant differences. Mean values and standard deviation were calculated for all parameters. The analysis of variance test (ANOVA) was employed at a significance level of 5%, which was followed by the Duncan test (*p* < 0.05) for post hoc comparisons. In the food neophobia study, a *t*-test (*p* < 0.05) was performed to compare sensory results between neophobic and non-neophobic consumers. All statistical analyses were conducted using SPSS software, version 24.0 for Windows.

## 3. Results

### 3.1. Physical Parameters

As a first analysis, the physical parameters such as color or texture were analyzed, since these parameters may play an important role and influence consumer acceptability. The results obtained regarding color parameters (L*: lightness; a*: red–green component; b*: yellow–blue component; C*: chroma) are shown in Table 2.

The lightness values are lower in all the samples elaborated with seed oils compared to the control sample, indicating slightly darker colors. This is likely due to the presence of pigments in the oils replacing the typically used pork fat in dry-cured sausage production, which is known for its white tones. Lightness in meat and meat products may depend on factors such as water retention capacity and fat content [41], but in this case, it also can be influenced by pigments contained in the oils used.

All samples exhibit positive values in the red–green and yellow–blue components, as previously observed in other studies [42]. However, the inclusion of different oils is associated with changes in color compared to the control sample. The most notable differences were found in the samples in which pumpkin oil was included due to the greater variety of pigments contained in this oil. The color of pumpkin oil can range from dominant orange to dark brown due to the presence of β-carotene, α-carotene, and lutein [43], but its pigment content is more intricate. This oil is rich in chlorophylls, including chlorophyll a and chlorophyll b, which contribute to its greenish color [44], imparting green tones and significantly affecting the final color of the samples prepared with pumpkin oil, leading to a decrease in the value of the a* component.

These pigments not only contribute to the visual appearance of the sausages but also provide potential health benefits due to their antioxidant properties [44,45].

Texture is another important physical parameter of food that can lead to consumer rejection. Table 3 shows the results obtained regarding the texture parameters considered in the TPA (hardness, springiness, cohesiveness, and chewiness) of the different formulations of dry-cured sausages produced.

The texture of the samples where seed oils were included showed similar values to the control sample in all the parameters considered except for cohesiveness, where the control sample significantly differed from all the others. Moreover, it is observed that the values of cohesiveness decrease when higher proportions of seed oils are used. Fat plays a crucial role in the cohesion of cured meat products, since it acts as a binding agent, helping to unite the ingredients and create a cohesive structure. During the curing process, fat melts and distributes evenly within the product, providing a smooth and juicy texture [7]. Thus, the fat used to substitute pork fat should have a similar texture and characteristics to avoid cohesion problems during the production of cured meat products. Indeed, while the texture of oleogels may resemble that of animal fats, their behavior within the product is distinct, leading to lower evaluations in the analysis. The differences in behavior can be attributed to various factors, including the ability of animal fats to interact more effectively with the other components of the formulation, which contributes to the overall sensory experience and cohesiveness in the final product. The unique properties of oleogels and their interactions with the surrounding matrix require careful consideration and optimization to achieve results comparable to those obtained with traditional animal fats.

Among the remaining parameters, no significant differences were found, as they presented very similar values, indicating a comparable texture regardless of the type or percentage of textured seed oil used, except for samples made with chia oil at the maximum replacement percentages (75% and 100%), which showed lower hardness values. This observation can be attributed to the softer texture of the oleogel containing a higher proportion of unsaturated fatty acids, particularly linolenic acid, which influences the overall hardness of the product. Similar findings were reported in previous studies [42]. Regarding springiness, no significant differences were detected among the samples except for sausages elaborated with pumpkin oil (50 and 75%) and melon oil (75%), although a previous study on sausages observed that a higher proportion of animal fat contributes to increase this parameter [46].

### 3.2. Proximate Analysis

Proximate analysis provides valuable information about the composition and nutritional quality in food, since it is involved in the determination of nutritional components such as ashes, fat, proteins, and carbohydrates. The results obtained from the proximate analysis in the dry-cured sausages are shown in Table 4. The partial or total replacement of pork fat by textured seed oils implies a reduction in the fat content of the product, since this texturing consists of an emulsion of oil with inulin and guar gum in which water is included as an ingredient [47]. Therefore, the higher the percentage of replacement of pork fat by textured oils, the greater the fat reduction in the product. This fact aligns with the current preference of consumers, who prioritize low-fat food options.

When considering the proximate analysis of the products on a dry basis, the first outcome observed upon incorporating seed oils into the *salchichón* formulation is a reduction in the overall fat content. This is attributed to the fact that seed oils undergo texturization with water, resulting in a decreased quantity of fat in the overall recipe. Therefore, the reduction in fat content is more evident when the replacement of animal fat by seed oil is complete. This result agrees with the current trends to reduce dietary fat to prevent or reduce the risk of chronic metabolic disease like obesity, diabetes or certain types of cancer [48]. This reduction in fat content allows the other nutrients to occupy a larger percentage of the overall composition, resulting in increased levels of ashes and total protein content. This effect is particularly noticeable in cases where the total replacement of fat is implemented regardless of the type of seed used.

Moreover, the reduction in the fat content of the sausages is reflected in a decrease in the energy value, which is also significantly greater with a higher percentage of substitution.

### 3.3. Fatty Acid Pattern

The reduction in total fat in dry-cured sausages elaborated with textured seed oils is accompanied by changes in the fatty acid pattern, since seed oils are rich in unsaturated fatty acids. Thus, the total substitution of pork fat by vegetable oils leads to an increase in polyunsaturated fatty acids in the dry-cured sausages (Table 5).

The high proportion of linolenic acid in chia oil makes this oil a good substitute to replace pork fat and reduce the proportion of saturated fatty acids in meat products [20]. The inclusion of chia oil in the formulation of dry-cured sausages is reflected in the presence of a high proportion of linolenic acid in these samples. Linolenic acid is an essential fatty acid from the omega-3 (ω-3) family, which has been found to be associated with anti-inflammatory processes that are beneficial for health [49].

On the other hand, the other seeds used (poppy, pumpkin, and melon) contain a greater proportion of linoleic acid, which is reflected in the significantly higher proportion in the sausages made with their textured oils. Linoleic acid is another essential fatty acid (ω-6) which may also show positive effects on health, since previous studies have shown that higher tissue and circulating concentrations of linoleic acid are associated with a decreased risk of cardiovascular events [50,51].

In any case, all the samples of *salchichón* made with textured seed oils showed a lower proportion of saturated fatty acids, which aligns with current trends of reducing these saturated fats in the diet, although there is currently some controversy regarding their harmful effects on health.

### 3.4. Sensory Analysis

**Figure 1:**
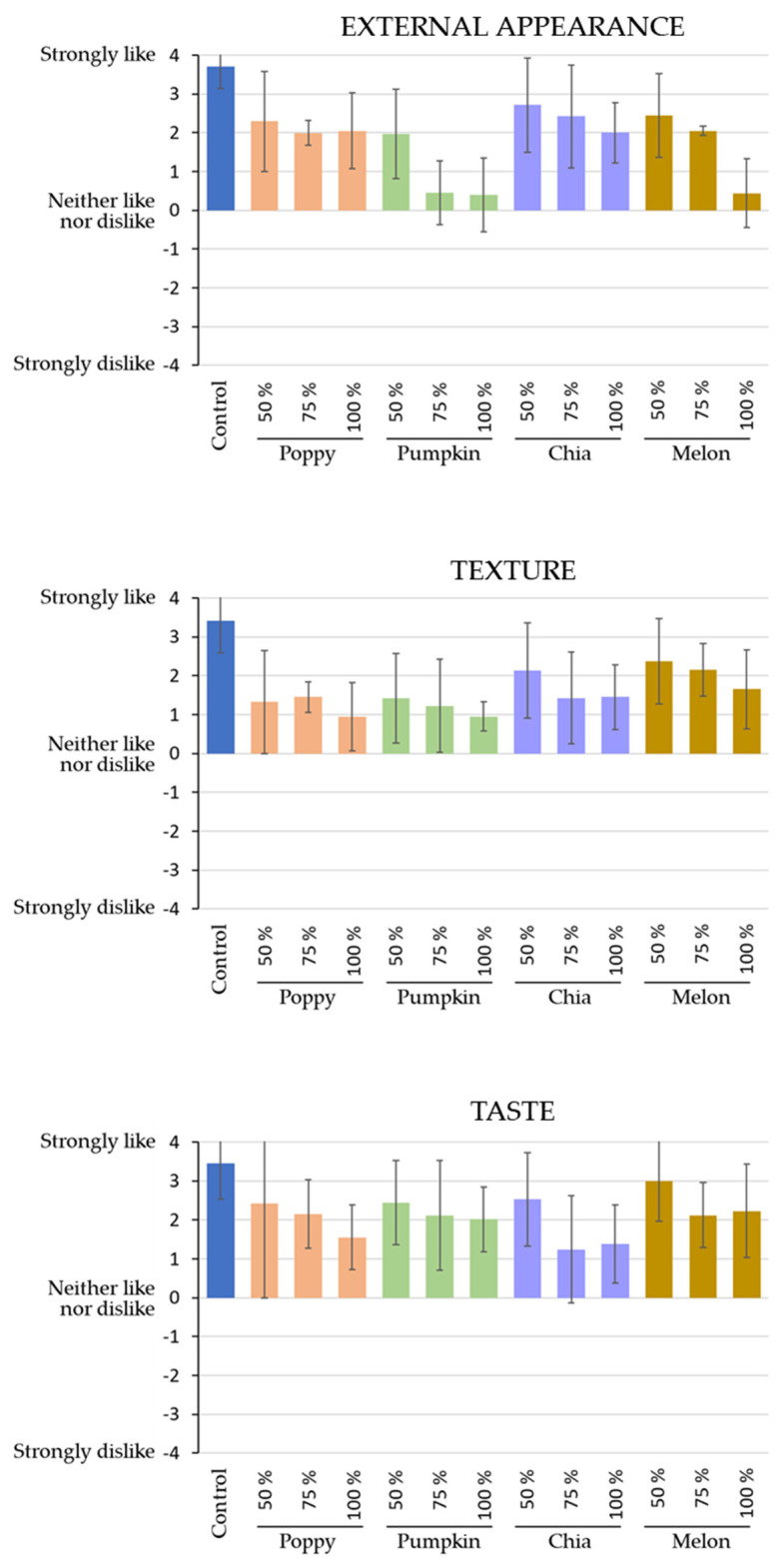
Average values of the sensory evaluation of the dry-cured sausages made with textured seed oils for the parameters: external appearance, texture, and taste.

Sensory analysis is a crucial aspect in the evaluation of novel foods due to its ability to provide valuable insights into the consumer perception of these foods, which is essential for ensuring their acceptability. This evaluation helps identify any sensory characteristic that may impact consumer acceptance, providing useful information to improve the development process of the product. The results obtained from the sensory analysis of dry-cured sausages elaborated with textured seed oils are shown in Figure 1. When a main ingredient is replaced, the product characteristics may be affected, which leads to changes in sensory properties and influences the consumers’ perception. In this sense, the best values for all the parameters evaluated were obtained in the control sample with the original formulation that includes pork fat.

In terms of external appearance, it was observed that all samples prepared with the textured seed oils exhibited lower scores compared to the control, which differs from findings in previous studies [42]. This trend was particularly evident as the percentage of seed oil used increased. The control sample received the highest rating, showing a significant difference from the other samples. The least favored samples were those prepared with 75% and 100% pumpkin seed oil, as well as 100% melon seed oil, scoring close to the “neither like nor dislike” range (between 0 and 1). This could be attributed to the darker color of pumpkin seed oil, which adversely affects the color of the sausages, resulting in an unfavorable perception by consumers. Furthermore, the inclusion of seed oils led to a less cohesive texture, which also had a negative impact on the overall appearance and subsequently decreased the consumer acceptance of these products.

Regarding texture, once again, the control sample showed significant differentiation from the remaining samples, which was likely attributable to the reduced cohesiveness resulting from the incorporation of seed oils. Minimal variations were observed among the samples elaborated with seed oils regardless of the seed used. However, it is worth noting that higher percentages of textured seed oil corresponded to lower texture scores.

Regarding taste, once again, the control sample obtained the highest scores. It significantly differed from the rest of the samples, as all the sausage samples made with textured seed oils received lower ratings. However, all the samples evaluated had positive ratings, with values over 2, corresponding to “quite liked”. The only exception among the evaluated samples was the sausage made with 75% and 100% chia oil, and 100% poppy oil, which received a lower rating. The high content of linolenic acid (ω-3) in chia oil can result in particular odors and flavors in products made with this oil, leading to consumer rejection when used in large quantities. Although no comprehensive studies have specifically focused on characterizing the aromas of chia oil, from a sensory point of view, the flavor of chia oil is commonly described as “nutty” or “cream” [52]. It is not an unpleasant aroma, but generally, consumers do not expect to find it in meat products, and when the sensory analysis is conducted, it is negatively evaluated, leading to lower scores in terms of smell or taste, which is similar to other studies carried out with different products where chia oil has been included [53].

Nevertheless, it is important to highlight that even though the values of the samples produced with seed oils were lower compared to the control sample, it is noteworthy that all evaluations fell within the positive range. This indicates a high level of acceptability among consumers, emphasizing their favorable perception of the product.

### 3.5. Analysis of Consumers’ Acceptance of the Sausages and Willingness to Pay

Table 6 shows the results obtained in the analysis of consumer preferences using ML. As anticipated, the findings demonstrate that a higher price exerts a significant adverse impact on consumers’ willingness to purchase sausage [54]. Table 7 shows the willingness to pay estimated in euros/500 g of product.

The impact of the pig breed (100% Iberian pig) on purchasing attitude is also positive, showing a WTP of 1.244 €/500 g. Products obtained from Iberian pigs are expected to be higher quality, tastier and healthier than pork products from white commercial breeds [36]. The influence of the animal welfare label on purchasing attitudes is also positive, indicating a willingness to pay (WTP) of 0.655 €/500 g. Overall, consumers in most countries exhibit significant concerns regarding the ethical aspects of animal production systems and their impact on animal welfare [55]. Several previous studies have further demonstrated that consumers perceive products derived from production systems that prioritize animal welfare not only as healthier but also safer, more flavorful, authentic, and traditional [56,57].

In contrast to the previous attributes, the presence of the pumpkin seed oil label has a detrimental effect on consumers’ purchasing attitudes. Consequently, a negative WTP value is observed (−3.000 €/500 g). A similar study by Tarjuelo et al. [58] found that consumers’ WTP for burgers elaborated using seed oils was also negative (−0.2831 €/pack of two burgers). This may be attributed to consumers perceiving seeds as fiber-rich and less appetizing food options [59,60]. It must be considered that some consumer segments tend to actively reject unfamiliar foods [23]. However, the standard deviation coefficient for the use of pumpkin seed oil is very strong (value 2.278) and highly significant. The latter demonstrates a significant variation in preference for this attribute despite the overall negative preference within the group. For that reason, further research to find consumer segments open to the use of pumpkin seed oils in the elaboration of sausages should be developed.

## 4. Conclusions

The substitution, both partial and total, of animal fat by textured seed oils (emulsified with inulin and guar gum) in dry-cured sausages causes changes in their physical characteristics mainly in terms of their texture, since less cohesive sausages are produced due to the role played by fat in these foods.

Likewise, there are changes in its nutritional characteristics where the lower fat content stands out due to the water content of the textured oil regardless of the oil used, which implies an increase in the amount of protein and total carbohydrates. This change is accompanied by a reduction in the total energy value.

The sausages made with chia oil have a higher proportion of linolenic acid (ω-3), while the sausages made with oils from the rest of the seeds stand out for the increase in linoleic acid (ω-6). These changes are more significant when fat replacement occurs completely.

When the sensory analysis was performed, although the values obtained for the external aspect, texture and taste of the dry-cured sausages made with textured seed oils were lower than the control sample elaborated with pork fat, all the values were positive, indicating a good degree of acceptance by the consumer judges.

Future studies should include a longer storage period for the sausages to investigate the stability of these new formulations over time.

## Figures and Tables

**Table 1:** Attributes and level used in the study.

Attributes	Levels
Animal welfare	No information
	Animal welfare label
Breed	50% Iberian pig
	100% Iberian pig
Price	5.50 €/500 g
	8.00 €/500 g
	10.50 €/500 g
Pumpkin seed oil	No information
	“With pumpkin seed oil” label

**Table 2:** Results obtained for color parameters in the samples elaborated with textured seed oils.

Sample	L*	a*	b*	C*
Control	50.93 ^a^ ± 2.86	17.51 ^a^ ± 1.47	8.24 ^b^ ± 1.07	19.35 ^a^ ± 1.68
Poppy 50%	40.48 ^b^ ± 3.84	14.98 ^b^ ± 1.99	8.05 ^b^ ± 0.88	17.00 ^b^ ± 2.01
Poppy 75%	35.21 ^c^ ± 3.91	14.12 ^b^ ± 1.62	8.66 ^b^ ± 1.91	16.56 ^b^ ± 2.32
Poppy 100%	33.12 ^c^ ± 2.92	14.10 ^b^ ± 1.40	8.46 ^b^ ± 1.09	16.44 ^b^ ± 1.92
Pumpkin 50%	40.23 ^b^ ± 1.86	10.46 ^c^ ± 1.41	8.50 ^b^ ± 1.50	13.48 ^c^ ± 1.74
Pumpkin 75%	40.09 ^b^ ± 3.55	9.82 ^c^ ± 0.56	10.56 ^a^ ± 1.65	14.42 ^c^ ± 1.20
Pumpkin 100%	40.84 ^b^ ± 1.98	9.79 ^c^ ± 1.52	9.16 ^ab^ ± 1.13	13.40 ^c^ ± 1.44
Chia 50%	38.22 ^bc^ ± 3.47	14.03 ^b^ ± 1.70	6.17 ^c^ ± 1.19	14.42 ^c^ ± 1.98
Chia 75%	40.12 ^b^ ± 3.73	13.94 ^b^ ± 1.73	6.60 ^c^ ± 1.56	15.32 ^bc^ ± 2.17
Chia 100%	35.97 ^c^ ± 3.40	13.66 ^b^ ± 1.50	7.61 ^bc^ ± 1.14	15.60 ^bc^ ± 1.74
Melon 50%	38.76 ^bc^ ± 2.52	14.39 ^b^ ± 1.70	8.28 ^b^ ± 1.37	16.36 ^b^ ± 2.08
Melon 75%	38.62 ^bc^ ± 2.30	14.12 ^b^ ± 1.73	8.89 ^b^ ± 1.36	16.68 ^b^ ± 2.04
Melon 100%	36.78 ^bc^ ± 3.40	13.54 ^b^ ± 1.22	8.36 ^b^ ± 1.75	15.91 ^bc^ ± 1.51

^a,b,c^ Different letters in the same column indicate statistical differences (*p* < 0.05).

**Table 3:** Results obtained for texture parameters in the samples elaborated with textured seed oils.

Sample	Hardness (N)	Springiness	Cohesiveness	Chewiness (N)
Control	497.13 ^a^ ± 70.99	0.71 ^a^ ± 0.04	0.85 ^a^ ± 0.05	316.62 ^bc^ ± 220.97
Poppy 50%	507.75 ^a^ ± 60.30	0.67 ^ab^ ± 0.03	0.68 ^b^ ± 0.03	342.35 ^a^ ± 33.88
Poppy 75%	472.87 ^a^ ± 31.72	0.66 ^ab^ ± 0.11	0.59 ^c^ ± 0.03	278.43 ^c^ ± 30.44
Poppy 100%	462.80 ^a^ ± 31.72	0.66 ^ab^ ± 0.16	0.52 ^c^ ± 0.01	258.44 ^c^ ± 30.66
Pumpkin 50%	513.84 ^a^ ± 38.87	0.60 ^b^ ± 0.06	0.68 ^b^ ± 0.07	351.67 ^a^ ± 53.72
Pumpkin 75%	492.72 ^a^ ± 62.87	0.61 ^b^ ± 0.06	0.65 ^b^ ± 0.02	321.62 ^bc^ ± 36.17
Pumpkin 100%	477.67 ^a^ ± 99.70	0.68 ^ab^ ± 0.15	0.66 ^b^ ± 0.04	318.86 ^bc^ ± 77.73
Chia 50%	521.67 ^a^ ± 31.26	0.65 ^ab^ ± 0.12	0.66 ^b^ ± 0.01	343.51 ^a^ ± 14.22
Chia 75%	336.44 ^b^ ± 13.51	0.65 ^ab^ ± 0.06	0.64 ^bc^ ± 0.02	217.99 ^c^ ± 93.69
Chia 100%	395.21 ^ab^ ± 82.42	0.65 ^ab^ ± 0.05	0.59 ^c^ ± 0.02	233.07 ^c^ ± 51.41
Melon 50%	478.40 ^a^ ± 43.68	0.68 ^ab^ ± 0.06	0.71 ^b^ ± 0.05	341.37 ^a^ ± 36.30
Melon 75%	527.63 ^a^ ± 12.14	0.62 ^b^ ± 0.05	0.62 ^bc^ ± 0.01	328.29 ^bc^ ± 14.80
Melon 100%	444.77 ^a^ ± 95.60	0.65 ^ab^ ± 0.07	0.59 ^c^ ± 0.02	261.25 ^c^ ± 53.90

^a,b,c^ Different letters in the same column indicate statistical differences (*p* < 0.05).

**Table 4:** Results obtained from the proximate analysis of the dry-cured sausages elaborated with textured seed oils.

Sample	Ashes (%)	Protein (%)	Fat (%)	Total Carbohydrates (%)	Energy Value (Kcal)
Control	6.92 ^c^ ± 0.24	41.50 ^c^ ± 1.72	46.31 ^a^ ± 1.35	5.27 ^b^ ± 0.32	604 ^a^ ± 2.60
Poppy 50%	7.57 ^c^ ± 0.37	47.06 ^b^ ± 2.04	43.32 ^a^ ± 2.19	2.05 ^c^ ± 0.21	586 ^a^ ± 3.41
Poppy 75%	9.17 ^b^ ± 0.75	54.38 ^a^ ± 2.46	31.24 ^bc^ ± 1.27	5.22 ^b^ ± 0.24	520 ^c^ ± 2.70
Poppy 100%	9.21 ^b^ ± 0.81	55.25 ^a^ ± 1.87	30.43 ^c^ ± 1.09	5.11 ^b^ ± 0.43	515 ^c^ ± 1.80
Pumpkin 50%	8.74 ^b^ ± 0.62	52.88 ^ab^ ± 1.12	34.86 ^b^ ± 1.56	3.52 ^c^ ± 0.32	539 ^b^ ± 4.23
Pumpkin 75%	9.15 ^b^ ± 0.42	49.63 ^b^ ± 1.63	34.52 ^b^ ± 1.78	6.71 ^ab^ ± 0.40	536 ^b^ ± 2.81
Pumpkin 100%	9.96 ^a^ ± 0.65	52.81 ^ab^ ± 1.35	31.50 ^bc^ ± 1.63	5.73 ^b^ ± 0.33	518 ^c^ ± 1.96
Chia 50%	9.07 ^b^ ± 1.02	49.50 ^b^ ± 1.88	37.15 ^b^ ± 1.25	4.28 ^b^ ± 0.20	549 ^b^ ± 2.04
Chia 75%	9.13 ^b^ ± 1.15	51.38 ^b^ ± 2.01	33.67 ^b^ ± 1.17	5.82 ^b^ ± 0.38	532 ^bc^ ± 2.74
Chia 100%	9.65 ^ab^ ± 0.96	55.00 ^a^ ± 2.46	27.15 ^c^ ± 1.06	8.20 ^a^ ± 0.51	497 ^c^ ± 3.16
Melon 50%	8.97 ^b^ ± 0.85	49.69 ^b^ ± 1.23	35.41 ^b^ ± 1.91	5.93 ^b^ ± 0.17	541 ^b^ ± 2.92
Melon 75%	8.49 ^b^ ± 0.79	51.19 ^b^ ± 1.52	37.46 ^b^ ± 1.85	2.86 ^c^ ± 0.12	553 ^b^ ± 3.40
Melon 100%	8.99 ^b^ ± 0.98	56.19 ^a^ ± 2.23	28.04 ^c^ ± 1.36	6.78 ^ab^ ± 0.36	504 ^c^ ± 2.60

^a,b,c^ Different letters in the same column indicate statistical differences (*p* < 0.05).

**Table 5:** Content of the main fatty acids in the dry-cured sausages elaborated with seed oils.

Sample	Palmitic Acid C16:0	Stearic Acid C18:0	Oleic Acid C18:1	Linoleic Acid C18:2	Linolenic Acid C18:3
Control	25.1 ^a^ ± 0.85	13.7 ^a^ ± 0.73	46.9 ^a^ ± 0.44	8.0 ^c^ ± 0.44	0.55 ^c^ ± 0.12
Poppy 50%	23.1 ^b^ ± 1.41	12.3 ^ab^ ± 0.04	40.9 ^ab^ ± 0.37	17.7 ^b^ ± 0.19	0.74 ^c^ ± 0.11
Poppy 75%	20.0 ^c^ ± 1.38	9.2 ^c^ ± 0.49	36.1 ^b^ ± 0.65	29.0 ^a^ ± 1.01	0.79 ^c^ ± 0.20
Poppy 100%	20.9 ^c^ ± 1.62	9.3 ^c^ ± 0.87	33.6 ^bc^ ± 1.81	31.2 ^a^ ± 1.02	0.83 ^c^ ± 0.33
Pumpkin 50%	23.2 ^b^ ± 1.29	11.8 ^b^ ± 0.92	41.1 ^ab^ ± 1.63	17.8 ^b^ ± 0.52	0.82 ^c^ ± 0.21
Pumpkin 75%	22.2 ^bc^ ± 1.33	11.3 ^b^ ± 0.65	39.4 ^b^ ± 1.22	21.4 ^b^ ± 1.45	1.26 ^c^ ± 0.30
Pumpkin 100%	21.0 ^c^ ± 0.99	10.1 ^c^ ± 0.88	37.9 ^b^ ± 1.34	25.6 ^b^ ± 1.20	1.22 ^c^ ± 0.36
Chia 50%	19.7 ^c^ ± 1.37	10.1 ^c^ ± 0.72	35.1 ^b^ ± 1.32	16.4 ^b^ ± 0.88	14.28 ^b^ ± 0.11
Chia 75%	19.1 ^c^ ± 1.45	10.0 ^c^ ± 1.01	34.0 ^bc^ ± 1.45	16.6 ^b^ ± 0.66	15.21 ^b^ ± 0.62
Chia 100%	17.5 ^c^ ± 1.37	8.5 ^c^ ± 1.55	29.0 ^c^ ± 0.99	18.0 ^b^ ± 1.07	23.36 ^a^ ± 0.23
Melon 50%	22.2 ^bc^ ± 0.99	12.1 ^ab^ ± 1.27	38.4 ^b^ ± 0.97	21.9 ^b^ ± 1.01	0.78 ^c^ ± 0.31
Melon 75%	20.0 ^c^ ± 1.14	10.1 ^c^ ± 1.10	35.9 ^b^ ± 1.22	27.9 ^a^ ± 1.01	0.86 ^c^ ± 0.21
Melon 100%	18.1 ^c^ ± 1.70	9.3 ^c^ ± 0.22	34.3 ^bc^ ± 1.67	33.7 ^a^ ± 1.22	0.85 ^c^ ± 0.36

^a,b,c^ Different letters in the same column indicate statistical differences (*p* < 0.05).

**Table 6:** Estimated parameters for ML model.

Attribute	Group Average		Individual Variation	
	Estimate (S.E.)	*p*-Value	Std. Dev.	*p*-Value
Price	−0.322 (0.044)	0.000 ***	0.242	0.000 ***
Animal welfare	0.655 (0.134)	0.000 ***	0.579	0.016 *
Pumpkin seed oil	−0.963 (0.274)	0.000 ***	2.278	0.000 ***
Breed	1.244 (0.225)	0.000 ***	1.548	0.000 ***
N. obs	2496			
Wald chi2	248.81			
Prob > chi2	0.0000			
logL	−657.37			
df	9			
AIC	1332.74			
BIC	1385.14			

*,*** indicate significant effects at 0.05 and 0.001 levels, respectively. S.E.: standard error; N. obs: number of observations; Wald chi2: Wald test; logL: log likelihood function; df: degrees of freedom; AIC: Akaike’s information criterion; BIC: Bayesian information criterion.

**Table 7:** Willingness to pay (WTP) estimated in euros.

Attribute	WTP (€/500 g)
Animal welfare	2.00494
Pumpkin seed oil	−2.99528
Breed	3.86924

## Data Availability

The data presented in this study are available on request from the corresponding author.
